# Computational neuroscience: a frontier of the 21^st^ century

**DOI:** 10.1093/nsr/nwaa129

**Published:** 2020-06-12

**Authors:** Xiao-Jing Wang, Hailan Hu, Chengcheng Huang, Henry Kennedy, Chengyu Tony Li, Nikos Logothetis, Zhong-Lin Lu, Qingming Luo, Mu-ming Poo, Doris Tsao, Si Wu, Zhaohui Wu, Xu Zhang, Douglas Zhou

**Affiliations:** Center for Neural Science, New York University, USA; Center for Neuroscience, Key Laboratory of Medical Neurobiology of the Ministry of Health of China, School of Medicine, Zhejiang University, China; Department of Neuroscience and Department of Mathematics, Center for the Neural Basis of Cognition, University of Pittsburgh, USA; Université Claude Bernard Lyon 1, Inserm, Stem Cell and Brain Research Institute U1208, France; Institute of Neuroscience, State Key Laboratory of Neuroscience, Chinese Academy of Sciences, CAS Center for Excellence in Brain Science and Intelligence Technology, China; Shanghai Center for Brain Science and Brain-Inspired Technology, China; Institute of Neuroscience, State Key Laboratory of Neuroscience, Chinese Academy of Sciences, CAS Center for Excellence in Brain Science and Intelligence Technology, China; Shanghai Center for Brain Science and Brain-Inspired Technology, China; Institute of Neuroscience, State Key Laboratory of Neuroscience, Chinese Academy of Sciences, CAS Center for Excellence in Brain Science and Intelligence Technology, China; Division of Arts and Sciences, and NYU-ECNU Institute of Cognitive Neuroscience, NYU Shanghai, China; School of Biomedical Engineering, Hainan University, China; Institute of Neuroscience, State Key Laboratory of Neuroscience, Chinese Academy of Sciences, CAS Center for Excellence in Brain Science and Intelligence Technology, China; Shanghai Center for Brain Science and Brain-Inspired Technology, China; Division of Biology and Biological Engineering, California Institute of Technology, USA; Howard Hughes Medical Institute, USA; School of Electronics Engineering and Computer Science, IDG/McGovern Institute for Brain Research, PKU-Tsinghua Center for Life Sciences, Peking University, China; College of Computer Science and Technology, Zhejiang University, China; Institute of Brain-Intelligence Science and Technology, Zhangjiang Lab, China; Shanghai Center for Brain Science and Brain-Inspired Technology, China; School of Mathematical Sciences, MOE-LSC, and Institute of Natural Sciences, Shanghai Jiao Tong University, China

## THEORY IN NEUROSCIENCE

The human brain is a biological organ, weighing about three pounds or 1.4 kg, that determines our behaviors, thoughts, emotions and consciousness. Although comprising only 2% of the total body weight, the brain consumes about 20% of the oxygen entering the body. With the expensive energy demand, the brain enables us to perceive and act upon the external world, as well as reflect on our internal thoughts and feelings. The brain is actually never at ‘rest’. Brain activities continue around the clock, ranging from functions enabling human–environment interactions to housekeeping during sleep, including processes such as synaptic homeostasis and memory formation. Whereas one could argue that sciences in the last century were dominated by physics and molecular biology, in the current century one of our major challenges is to elucidate how the brain works. A full understanding of brain functions and malfunctions is likely the most demanding task we will ever have.

To accelerate breakthroughs in neuroscience, the US Brain Initiative was launched in 2013. An advisory committee to the Director of the National Institutes of Health was charged to chart a roadmap for this initiative in consultation with the neuroscience community. Their report [1] identified seven priorities, mostly focused on technological developments (the report's title is Brain Research through Advancing Innovative Neurotechnologies (BRAIN)). While brain research is fundamentally an empirical scientific field driven by experimental tools, priority #5 stresses theory and computational modeling: ‘Rigorous theory, modeling, and statistics are advancing our understanding of complex, nonlinear brain functions where human intuition fails. New kinds of data are accruing at increasing rates, mandating new methods of data analysis and interpretation. To enable progress in theory and data analysis, we must foster collaborations between experimentalists and scientists from statistics, physics, mathematics, engineering, and computer science.’ These recommendations have served the neuroscience community well. For instance, neurophysiologists used to record from one neuron at a time in behaving animals; the invention of the Neuropixel probe has enabled recording tens of thousands of neurons across multiple brain regions in behaving animals. Given the complexity of neural systems and the enormous amount of data from experimental studies, recommendation #5 recognized the importance of theory and computational models in neuroscience.

Why? The brain is composed of a vast number of neurons and characterized by ultra-high complexity of structural connectivity, all of which change and evolve in response to experience. Information related to sensors and effectors is processed in a parallel as well as recurrent fashion. The connectivity between different hierarchical levels is often bidirectional, its effectiveness is continuously reconfigured according to behavioral demands as well as controlled by neuromodulatory systems. In mathematical physics, structures with similar properties of the brain are termed adaptive complex dynamic systems. For such systems, the behavior of the whole cannot necessarily be reduced to, or predicted from, the activity of its components. Complex physical systems are commonly characterized by self-organization of a rich repertoire of dynamical states, and possible transitions to so-called catastrophic states of abnormal behavior. The structural and functional organization of complex dynamical systems is overwhelming.

Theory and computational modeling play an increasingly important role in tackling this challenge. First, massive data from brain connectomics, transcriptome and neurophysiology increasingly demand novel analysis tools being developed by theorists. Second, the brain systems are too complex to comprehend by experiments and intuition alone. For instance, cortical areas interact with each other through connection loops; optogenetic inactivation of one area may have impacts on multiple brain regions that are hard to understand and insights can be gained by computational modeling as a complementary platform. Third, theory and modeling, in concert with experimentation, are needed to advance our understanding of how the brain works across spatiotemporal scales, from molecules to neural circuits, and to functions and behavior. Theory goes beyond models, striving for generalization and universal principles. Data analysis, modeling and theory closely interact with each other, and in tandem with laboratory experiments.

## EPISTEMOLOGY OF COMPUTATIONAL NEUROSCIENCE

Neuroscience is tremendously diverse, and how to best advance theory remains a matter of debate. It has been argued that instead of a monolithic framework like Newtonian mechanics in physics, a more plausible approach is to seek a mosaic unity of neuroscience. One pragmatic view on theory is that science is problem-solving [2]. Neuroscience is full of puzzles, and a theory is validated by its ability to explain observed phenomena associated with brain functions, such as how color is perceived, how a choice among multiple options is made or what is the brain mechanism of autism. The degree of success of a mathematical model can be measured by its ability to account for an increasing amount of empirical data, its simplicity and generalizability for novel testable predictions.

There are three types of modeling approaches [3]. First, *descriptive models* are designed to quantitatively characterize experimental data. Signal processing algorithms and stochastic process models for neuronal spike trains belong to this category, as do linear filter models of sensory neurons, or population coding and decoding algorithms. Second, *normative theories* aim at explaining brain processes at the functional level. For instance, Horace Barlow proposed decorrelation, a computation that renders neural coding of sensory information more efficient by reducing redundancy in stimulus inputs, for understanding multiple aspects of adaptation in early sensory systems. Statistical Bayesian inference theory argues that neural coding and processing of sensory stimuli depends on the organism's prior knowledge about the environment, hence can be optimized based on the prior probability distribution of the sensory input. Third, *mechanistic models*, also called *biologically-realistic models*, are constructed based on the two pillars of neuroscience: neuroanatomy (cell types, connectivity) and neurophysiology (from biophysics of neurons and synapses to neural population activity during behavior).

It is sometimes said that top-down theories are concerned with uncovering computational principles, whereas bottom-up realistic models deal with biological implementations. Although this distinction is useful for certain purposes, it should not be perceived in terms of one being superior to or more fundamental than another. The field has entered a new era when computational theories and biologically constrained models are integrated for understanding across levels.

## PAST, PRESENT AND FUTURE CHALLENGES

Modern computational neuroscience builds on two traditions. Neurophysiology is one of the most quantitative branches of biology, exemplified by the seminal Hodgkin and Huxley model of action potentials [4], influential mathematical models for neural population dynamics and learning and memory [5]. The second root is experimental psychology and computer science, focusing on information processing and learning, illustrated by artificial neural networks in the 1960s and learning algorithms at the origin of today's revolution in artificial intelligence. Pioneering works notwithstanding, computational neuroscience was officially born as a field at the end of the 1980s. In 1988 an article as a manifesto of the nascent field was published [6], and the Methods in Computational Neuroscience summer school was inaugurated at the Marine Biological Laboratory in Cape Cod near Boston. Over the past three decades, computational neuroscience has matured and advanced on multiple fronts [3,7].

Computational neuroscience has grown through close interactions with empirical research. For instance, models of single neurons were built based on great strides of *in vitro* neurophysiology; *in vivo* experiments inspired models on how the mammalian primary visual cortex generates orientation selectivity or how central pattern generators underlie locomotion. Theory played a key role in discovering general principles, such as normalization. Increasingly models yield unexpected predictions that are confirmed by empirical observations. For instance, modeling efforts to explain irregular spiking activities of cortical neurons led to the concept of balanced excitation and inhibition in the cortex, which has then been amply supported by experiments and become a central tenet of neuroscience [8]. Yet another example is reinforcement learning theory, initially developed in computer science, which now plays a central role in understanding the brain mechanisms of reward-dependent decision behavior. Likewise, we have also made substantial progress in understanding subcortical regulation of affective behaviors such as fear, anger, disgust, empathy and love. Most recently, progress in machine learning approaches for vision have led to exciting new developments in understanding the brain mechanisms for visual object recognition.

Back in 1988, computational neuroscience initially focused on the early stages of sensory processing [6], because studies of the neural bases of higher cognitive functions were largely in the realm of psychology and outside of empirical neuroscience of that era. The situation has changed dramatically since then. We have gained a large body of knowledge on the brain mechanisms of cognitive functions such as working memory (the brain's ability to internally maintain and manipulate information in the absence of sensory stimulation), decision-making (choosing one among several options based on the expected outcome and under uncertainty), selective attention and executive control of flexible behavior [9]. Progress in these areas is not only exciting for basic research but also holds promise for clinical applications. Most psychiatric disorders implicate the same brain systems underlying cognitive functions and executive control of behavior, with the prefrontal cortex at its core. Therefore, elucidating circuit mechanisms of cognitive functions, in the prefrontal cortex and its associated areas including the posterior parietal cortex and basal ganglia, is expected to yield a solid biological foundation for diagnosis and therapeutic treatment of mental illness. This line of research has led to the emergence of the new field of computational psychiatry [10].

Neuroscientific studies of humans, psychiatric patients in particular, have been greatly empowered by functional magnetic resonance imaging (fMRI). Yet, neural activity can only be indirectly estimated from fMRI, mainly reflecting changes in metabolic energy demands. In addition, because of the relatively poor spatial and temporal resolution, such brain imaging measures cannot differentiate between input/output-specific processing and neuromodulation, between bottom-up and top-down signals, and they may occasionally confuse excitation and inhibition. A multimodal approach combining experimental with computational and theoretical methodologies is more necessary than ever for the study of brain functions and dysfunctions. A ‘must’ in such systems is the implementation of multimodal and multiscale approaches that provide data across different hierarchical levels at the same time. The nested architecture of such systems would further demand a common language across levels, from single neurons to microcircuits and brainwide networks.

Looking ahead, the fast-moving field of neuroscience promises opportunities and challenges. One significant development is the fruitful exchanges between the fields of computational neuroscience and artificial intelligence [11]. Machine learning has been increasingly used in data analysis and computational modeling in brain research. Conversely, the current framework of artificial intelligence has been largely limited to input–output mappings such as object recognition or language translation. Discoveries of the brain mechanisms of higher cognitive functions such as multi-tasking, planning and creativity, translated into mathematical algorithms by computational models, will influence the next generation of smart machines and robots.

So far, the most detailed mechanistic neuroscience models have been largely limited to local circuits. The game changer is the ongoing deluge of big data from single-cell resolution transcriptome, cell-type specific and brain-wide connectome, large-scale neurophysiology, and functional brain activity mapping. The technological advances and the enriched empirical data put demands on new theories and computational models for multi-regional, large-scale brain circuits. This is the central message of the newly published white paper about the second phase (2020–25) of the US Brain Initiative [12]. The document reiterated priority #5 as ‘Identifying Fundamental Principles: Produce conceptual foundations for understanding the biological basis of mental processes through development of new theoretical and data-analysis tools.’ It asserts: 

In BRAIN 2.0, more attention could be paid to integrating the work of quantitative scientists of various types with experimental neuroscientists. Fuller integration of theory can also guide experimental design and enhance the validity of model systems. At the conclusion of the BRAIN Initiative, advances in this area will bring together theory and experiment to solve profound and overarching questions central to systems neuroscience, which will ultimately explain how intricately connected networks of neurons acquire the ability to govern behaviors, thoughts, and memories.

## INFRASTRUCTURE, EDUCATION AND FUNDING SUPPORT

The field of computational neuroscience now constitutes a vibrant worldwide community. It is no longer the case that a top university has only one theorist in neuroscience; Columbia, New York University and Stanford have each recruited a cluster of 5–6 theory faculty members. University of Chicago, University of California at Davis and other places are planning to go in the same direction. There is also a critical mass of computational neuroscientists in France, Germany and Spain; and computational modeling is the central theme of the European Human Brain Project. A lacuna on this map is China, which nevertheless has tremendous potential in this frontier field (http://news.sciencenet.cn/sbhtmlnews/2010/8/235983.html?id=235983; http://news.sciencenet.cn/htmlnews/2015/11/332429.shtm). Impor-tantly, China has a huge reservoir of young talents trained in physics, mathematics, engineering and computer science, who are increasingly attracted to neuroscience. With the recent rapid developments in systems neuroscience, theory and computational modeling are beginning to be considered as a priority of Chinese neuroscience.

At the national level, China Brain Project (‘Brain Science and Brain-Inspired Intelligence Technology’) has been approved by the State Council as one of the Innovation 2030 Major Science and Technology Projects [13]. Computational neuroscience will play an important role within the framework of ‘one body’ (Neural Basis of Cognition) and ‘two wings’ (Brain Diseases and Brain-Machine Intelligence Technology). For understanding the neural basis of cognition, a large amount of structural and functional information obtained by mapping neuronal connections at all scales will require development of efficient computational algorithms and analytical tools for data management and mining. For brain disease diagnosis and intervention, realistic modeling of physiological and pathological states of the brain and machine learning-assisted dissection of structural and functional abnormalities of the brain are invaluable for the early disease diagnosis and the evaluation of the efficacy of treatments. For brain-machine intelligence technology, the application of machine learning tools for coding and decoding neural signals will play a critical role in the brain-machine interface, and computational models and theories emerging from studying cognitive processes of the brain, from multi-sensory integration to decision-making and language processing, will inspire the development of the generation of machine learning algorithms and construction of neuromorphic computing devices and intelligent systems. Computational neuroscience serves to advance theory in basic brain research as well as psychiatry, and bridge from brains to machines. Therefore, it fits well with the stated ‘one body, two wings’ goal of the Chinese Brain Project.

To start a new subfield that demands sophisticated quantitative skills in neuroscience, it is essential to attract young talents from physics, mathematics, engineering and computer science and provide training opportunities to help their transitions to brain research. That mission was facilitated, starting in the early 1990s, by the establishment of Centers of Theoretical Neuroscience supported by the Sloan Foundation and later the Swartz Foundation. In the last three decades hundreds of young talents were trained in those centers, and many are now leaders of computational neuroscience. Similar programs were formed in Europe (including the German Network of Bernstein Centers and Gatsby Computational Neuroscience Center in England) and in Israel. We recommend that China establish two or more centers of theoretical neuroscience, with the dual goals of training young talents and coordinating computational brain research. These centers may be affiliated with elite universities or research institutes, supported by both the government and philanthropy. They would serve as hubs for the field across the country, as well as platforms for international collaboration in neuroscience.

It is worth keeping in mind that experimentalists and theorists may fail to provide the expected synergy due to a so-called ‘language-problem’. It is not unusual to experience allergic reactions of experimentalists facing the ‘non-understandable’ pages of coupled differential equations, nor is it strange that theorists may lose themselves in mathematics far removed from experimental reality. Potential future-centers in China should stress the multidisciplinary educational environment able to promote, encourage and improve direct communications between mathematically and experimentally oriented talents.

A second recommendation is to support summer schools in computational neuroscience. Training in such summer schools is—as mentioned above—crucial for both theorists transitioning from other fields unfamiliar with neuroscience and experimentalists who desire to learn modeling and theory. What is a ‘model’? What is a computer simulation? Can theory clarify hidden assumptions and suggest new experiments? As a matter of fact, an international summer course was launched in China 10 years ago, which has so far trained around 270 graduate students and postdoctoral fellows and is now well recognized (www.ccnss.org). Such summer programs should garner long-term support from the government as well as corporate and private sponsors.

Our third recommendation is to initiate a new funding program. Computational research must be conducted hand-in-hand with experimentation. Furthermore, reviewing theory grant applications needs expertise that may not be present in a traditional evaluation system in life sciences. These considerations led to the creation of the Collaborative Research in Computational Neuroscience (CRCNS) program jointly sponsored by the National Science Foundation and the National Institutes of Health in the US. A typical CRCNS grant application requires collaboration between an experimentalist and a theorist. Thus, a theorist does not build a model with published data; instead she or he starts with an experimentalist on formulating a scientific question and designing experiments to investigate that question. Through a back-and-forth process, theory and experimentation genuinely develop in an interactive, two-way street manner. Such a program in China would play a crucial role in fostering computational neuroscience. Furthermore, CRCNS has been expanded to joint programs with Germany, France, Israel and Japan. Once the Chinese program is established, it would be natural to consider an international collaboration with the CRCNS in the future.

To conclude, we express the urgency of mounting a serious and optimized effort to build computational neuroscience in China, which requires judicious planning. A mature National Neuroscience Program needs to incorporate theoretical general principles that integrate the biology of the brain and the psychology of the mind.

## Supplementary Material

nwaa129_Supplemental_File
