# Supplementary material for: Computational neuroscience: a frontier of the 21st century
Source: Natl Sci Rev. 2020 Jun 12;7(9):1418–22. doi: 10.1093/nsr/nwaa129 (PMC8288724; doi:10.1093/nsr/nwaa129)
Supplement: nwaa129_Supplemental_File [file nwaa129_supplemental_file.docx]

**计算神经科学：21世纪的前沿科学**

汪小京^1*^，胡海岚^2^ ，黄橙橙^3^ ，Henry Kennedy^4,5,6^，李澄宇^5,6^ ，Nikos Logothetis^5^，吕忠林^7^ ，骆清铭^8,9^ ，蒲慕明^5,6^ ，曹颖^10^ ，吴思^11^，吴朝晖^12^，张旭^13,6^ ，周栋焯^14^

^1^ 纽约大学神经科学中心，纽约，美国

^2^ 浙江大学神经科学中心，中国卫生部医学神经生物学重点实验室，医学院， 杭州，中国

^3^ 匹兹堡大学神经科学系和数学系，宾夕法尼亚州，美国； 认知神经基础研究中心，匹兹堡，宾夕法尼亚州，美国.

^4^ 法国里昂大学干细胞与大脑研究所， U1208, 69500 勒布朗，法国.

^5^ 中国科学院神经科学国家重点实验室神经科学研究所，中国科学院脑科学与智能技术研究中心，中国

^6^ 上海脑科学与类脑研究中心，上海，中国

^7^ 上海纽约大学文理学院,华东师范大学 - 纽约大学脑与认知科学联合研究中心（上海纽约大学)，中国

^8^ 海南大学生物医学工程学院，海口 570228，中国;

^9^ 华中科技大学苏州脑科学研究所，苏州 215125，中国

^10^ 加州理工学院生物与生物工程学系，加利福尼亚州; 霍华德·休斯医学研究所，美国.

^11^北京大学信息科学技术学院，IDG/麦戈文脑科学研究所，北京大学

-清华大学生命科学中心，中国

^12^ 浙江大学计算机科学与技术学院，杭州，中国

^13^ 张江实验室脑智能科学与技术研究所，上海，中国

^14^ 上海交通大学数学科学学院，科学工程计算教育部重点实验室，自然科学研究院，上海，中国

*联系人： [xjwang@nyu.edu](mailto:xjwang@nyu.edu)

2020年6月2日

**摘要：** 我们的大脑是一个极其复杂的生物动力学系统，它是我们个体行为、思想和情感的基础。鉴于神经系统的复杂性，从分子到神经回路再到功能的各个层面，理论和建模被越来越多地视为理解神经科学的必要条件。大脑连接组学和神经生理学的数据分析需要新的数学工具；计算机建模有助于探究大脑工作原理假说的细节并对特定的预测进行检验；理论工作可以帮助我们从数学上理解大脑运作的一般性原理。除了在基础研究中的贡献，计算神经科学还有助于为精神病学的发展建立坚实的理论基础，为脑科学与人工智能搭建合作的桥梁。在本文中，我们将讨论计算神经科学这一高度交叉学科领域的过去、现在、以及未来面临的挑战，并为推动中国计算神经科学的发展提出建议。

**神经科学中的理论**

人脑是一种生物组织，重约3磅或1.4千克，它决定了我们的行为，思想，情感和意识。尽管只占总体重的2％，大脑会消耗进入体内的氧气的大约20％。凭借着昂贵的能源消耗，大脑使我们能够感知外部世界并产生行动，形成内部思想和感受。大脑实际上永远不会处于“停息”状态。大脑昼夜不停地活动，实现从人与环境互动的日常功能到睡眠中的代谢功能调节，包括例如突触的内稳态平衡和记忆的产生。如果说上世纪的科学主要由物理和分子生物学主导，本世纪我们面临的主要挑战之一是阐明大脑的工作方式。全面了解大脑功能和功能障碍发生的机理可能是我们有史以来面临的最艰巨的科学任务。

为了加快神经科学领域的突破性进展，美国于2013年启动了脑计划， 由美国国立卫生研究院主任以及顾问委员会专门负责、与神经科学界协商、制定了计划的路线图。他们的报告 [1] 确定了七个优先事项，主要集中在技术发展上（该报告的标题是“Brain Research through Advancing Innovative Neurotechnologies”(BRAIN)）。尽管大脑研究从根本上是一个由实验工具驱动的实验科学领域，优先事项5强调了理论和计算模型的重要性：“严谨的理论、建模和统计方法正在推进我们对复杂的非线性大脑功能的理解；这是单凭人类的直觉所无法做到的。正以越来越快的速度产生的新数据也需要新的分析和解释的方法。为了在理论和数据分析方面取得进展，我们必须促进实验科学家与统计学、物理学、数学、工程学以及计算机科学领域的科学家之间的合作。”这些建议已经对神经科学的发展产生了非常积极的作用。例如，神经生理学家以前只能在行为动物上记录单个神经元，而Neuropixel探针的发明使得他们能够在行为动物的多个脑区同时记录上万个神经元。鉴于神经系统的复杂性和来自实验研究的大量数据，优先事项5确认了理论和计算模型在神经科学中的重要性。

为什么需要理论和计算模型？这是因为大脑由海量的神经元组成，其结构特点是连接的高度复杂性，而且这些连接会随着动物的经历而不断演化。与感知和行为相关的信息在大脑内以并行和循环方式进行处理，不同层次级别之间的神经元连接通常是双向的，它们的效用和连接强度会根据行为的需求不断地动态改变，并由神经调节系统来控制。在数学物理学中，具有类似大脑特征的结构被称为自适应复杂动态系统。对于这样的复杂系统，整体的行为并不一定能够约简到其组件的活动或者由组件的活动来预测。它们通常具有丰富的包含大量动力学状态的自组织行为，并且能够产生所谓的“灾难性”的异常状态。这类动力系统在结构和功能组织的复杂度远远超出我们的想象。

因此，理论和计算模型在理解复杂生物动态系统的挑战中正发挥着越来越重要的作用。首先，来自大脑连接组、转录组和神经生理学的海量数据越来越需要理论工作者开发和设计新的分析工具。其次，大脑系统过于复杂，无法仅仅通过实验和直觉来理解。例如，皮层区域通过环路连接进行交互作用，一个通过光遗传学失活的区域可能会对多个难以理解的大脑区域产生影响。 这种现象可以通过计算模型作为补充手段来获得更深刻的理解。再次，理论和模型与实验的紧密结合可以加深我们从跨时空的尺度对大脑工作原理的理解，从分子到神经回路到功能与行为的认识。重要的是，理论超越具体的模型，致力于创立普适性和通用的原则。数据分析、建模和理论可以相互促进，并与实验工作协同发展。

**计算神经科学的方法观**

神经科学研究本身是多样化的，如何最好地发展理论仍然是一个具有争议的问题。有一种观点认为，不应该寻找一个像牛顿力学在物理学中那样的整体框架，更可行的方法可能是发展能够组成神经科学的各个模块。一种实用性的观点认为科学是由解决问题而驱动发展的 [2]。神经科学充满了许多有待解答的谜题，一个理论的认可应该基于它是否能够解释与大脑功能相关的实验现象，比如大脑如何感知颜色、如何执行多选一的抉择、自闭症发生的大脑机制等。一个数学模型的成功程度可以通过它是否能够解释越来越多的经验数据、其形式的简单性、以及其对新的可检验的预测的普适性来衡量。

常用的建模方法有三种类型 [3]。第一，构建定量化地描述实验数据的描述性模型。神经元脉冲序列的信号处理算法和随机过程模型属于此类，感觉神经元的线性滤波器模型、以及群体神经元编码和解码算法也属于此类。第二，构建旨在功能水平上解释大脑工作过程的规范理论。例如，Horace Barlow提出的解释早期感觉系统自适应行为的去相关理论，即神经系统通过减少刺激输入信息的冗余性更有效地编码感觉信息；而贝叶斯统计推断理论认为神经编码和感觉刺激的处理依赖于生物体对环境的先验知识，可以根据感知的先验概率分布进行信息的优化加工。第三，构建基于神经科学的两大支柱：神经解剖学（细胞类型，连通性）和神经生理学（从神经元和突触的生物物理学到行为涉及的群体神经元活动）的生物仿真模型。

有些人认为自上而下的理论关注的是计算原理的发现，而自下而上的仿真模型关注的则是生物学的实现过程。尽管这种区分在某些场合是有用的，我们不应该理解为一种模型比另一种更优越或更基本。计算神经科学的发展已经进入了一个新时期，它需要通过结合计算理论和受生物约束的模型来实现对大脑工作原理的跨层次理解。

**过去、现在和未来的挑战**

现代计算神经科学建立在两种传统之上。其一是神经生理学，这是生物学中最定量的分支之一，以刻画动作电位的经典的Hodgkin 和Huxley模型 [4] 为代表，还包括其它描述神经元群体动力学和学习、记忆 [5] 的数学模型。其二是实验心理学和计算机科学，集中在信息处理和学习这两方面，如1960年代的感知器和成为当今人工智能革命起源的反向传播框架。这些都是早期开创性的工作，但计算神经科学作为一门独立的学科是在1980年代末才算正式诞生的。1988年是标志性的一年，同年关于这给此新兴领域的一篇宣言发表[6] 、“计算神经科学方法”暑期班在波士顿附近的海洋生物实验室开始举办。在过去的三十年中，计算神经科学已日趋成熟并且促成了多个领域的发展 [3,7]。

通过与实验研究的紧密互动，计算神经科学已获得越来越多的成果。例如，随着离体神经生理学实验取得的巨大进步，计算神经科学家建立了单个神经元的模型；载体实验则启发了有关哺乳动物初级视觉皮层方向选择性的模型，以及基于中枢模式发生器的运动模型。理论神经科学也促成了发现新原则，比如“除法归一化” （divisive normalization）。模型产生新概念的例子也越来越多。例如，为了解释皮层神经元不规则放电活动，数学模型提出了“均衡态” （balanced excitation and inhibition）的概念，后来得到了皮层实验的充分支持，成为神经科学领域的中心原则之一 [8]。另一个例子是最初在计算机科学领域发展起来的强化学习理论，，现在它在理解依赖于奖励的决策行为的大脑机制中起到了核心作用。同样，我们在理解情绪行为的皮层下调节机制方面也取得了实质性进展，包括恐惧、愤怒、厌恶、共情和爱等。近年来机器深度学习在视觉方面的应用，促进了我们在理解视觉目标识别的大脑机制方面取得了令人兴奋的新进展。

在1988年或更早，计算神经科学最初关注的是知觉信息处理的早期阶段 [6]，因为那时对更高级认知功能的研究主要集中在心理学，而不是实验神经科学领域。自那时以来，情况已经发生了巨大的变化。我们现在已经获得了有关认知功能的大脑机制的大量知识，例如工作记忆（大脑在没有感觉刺激的情况下内部维持和操控信息的能力）、决策（根据预计的结果从几个选项中选择一项）、注意、灵活行为的执行控制等 [9]。这些领域的进展不仅对于基础研究来说令人振奋，而且为临床应用也带来了希望。大多数精神疾病都牵涉到以前额叶皮层为核心的同一网络系统，这些系统是认知功能和行为执行控制的基础。因此，阐明前额叶皮层及其相关区域（包括后顶叶皮层和基底神经节）中认知功能的回路计算机制有望为诊断和治疗精神疾病提供坚实的生物学基础。这方面的研究促成了“计算精神病学”这个新领域的出现 [10]。

功能磁共振成像（fMRI）大大增强了对人类，包括精神病患者的神经科学的研究。但是， fMRI只能用来间接估计神经活动，因为它主要反映代谢能量需求的变化。此外，由于相对较低的空间和时间分辨率，这类大脑成像方法无法区分输入/输出特异性处理和神经调节，以及自下而上和自上而下的信号，并且有时会混淆兴奋和抑制。为了克服fMRI的局限性，我们必须将实验手段与计算模型、理论方法相结合起来，多模式研究大脑功能以及功能障碍。在大脑这样复杂的系统中，必须采用多模式和多尺度方法获取同一时间的跨层级数据，才能真正实现对其功能的理解。同时，这类系统的内在层级结构也需要一种“共同语言”来保证从单神经元到微电路到脑网络之间的信息交流。

展望未来，快速发展的神经科学领域充满机遇和挑战。一个重要的发展方向是计算神经科学和人工智能领域之间富有成果的交流 [11]。机器学习在大脑研究中的数据分析和计算建模中得到了越来越多的应用。相反，当前的人工智能框架在很大程度上仅限于实现输入-输出映射，例如物体识别或语言翻译。有关更高级的大脑认知功能机制的发现，如多任务、计划和创造力等，通过数学模型转换成算法，将对新一代智能机器和机器人产生重要影响。

迄今为止，最细致的神经科学机理模型大多局限于局部神经回路。不断涌现的来自单细胞分辨率转录组(single-cell resolution transcriptome )、细胞类型特异性全脑神经联接图谱 (cell-type specific and brain-wide connectome)、大规模神经生理学、以及功能性脑活动图谱的大数据，要求我们发展大尺度、多脑区的神经网络模型和理论。这也是新发布的第二阶段美国脑计划白皮书（2020年至2025年）的核心内容 [12]。该文件重申了优先事项5作为“识别基本原理：通过发展新的理论和数据分析工具，为理解心理过程的生物学基础提供概念性基础。” 它主张：“在BRAIN 2.0中，应加强多学科定量科学家与实验神经科学家的合作。与理论结合可以更好地指导实验设计并增强模型系统的有效性。在脑计划的完成时，该领域的进展将汇集理论和实验以解决系统神经科学中最至关重要，深刻并且全面的问题，最终解释复杂的神经网络如何获得控制行为、思想和记忆的能力”。

**基础设施、教育和经费支持**

计算神经科学领域已经成为了一个充满活力的国际社区。一所顶尖的大学不再只有一名理论神经科学家，哥伦比亚大学、纽约大学、斯坦福大学等都各自分别招募了5-6名理论神经科学教授。芝加哥大学、加州大学戴维斯分校和其他一些地方也正计划朝这同一方向发展。在法国、德国、西班牙等也有大量的计算神经科学家。计算建模是欧洲人脑计划的核心主题。尽管中国在这一前沿领域有着巨大的发展潜力 [13，14]， 目前还是一个空白。重要的是，中国拥有大量的在物理、数学、工程和计算机科学方面受过系统训练的青年人才，他们对神经科学也越来越感兴趣。随着近年来系统神经科学的迅速发展，理论和计算建模开始被作为中国神经科学优先考虑发展的方向。

在国家层面，中国脑计划（“脑科学和类脑研究”）已被国务院批准为“2030重大科技创新项目”之一 [15]。计算神经科学将在“一体”（认知的神经基础）和“两翼”（脑疾病和脑机器智能技术）的框架内发挥重要的作用。为了理解认知的神经基础，通过测量各种尺度的神经连接而获得的大量结构和功能信息将需要高效的算法和分析工具来进行数据管理和分析。在脑疾病的诊断和干预方面，脑部正常生理和病理状态的模型以及脑部结构和功能的机器学习分析对于脑疾病的早期诊断和治疗效果评估都非常重要。在脑机智能技术方面，应用机器学习对神经信号进行编码和解码将在开发脑机界面中发挥关键作用，而从研究脑的认知过程，多模态感觉整合到决策以及语言处理中产生的计算模型和理论，将激发机器学习新算法的产生以及神经形态计算设备和智能系统的构建。计算神经科学将推动基础脑科学研究以及精神病学理论的发展，并在大脑与机器之间架起桥梁。因此，它非常符合中国脑计划“一体两翼”的发展目标。

要启动一个新的需要复杂定量技能的神经科学的子领域，必须吸引物理学、数学、工程学和计算机科学领域的青年人才，并提供帮助他们转型到脑科学研究的培训机会。从1990年代初开始，多个由Sloan基金会和后来的Swartz基金会支持的理论神经科学中心的建立促成了这一使命。在过去的三十年中，这些中心培训了数百名青年人才，其中许多现在已经成为计算神经科学领域的领军人。欧洲（包括德国的Bernstein中心、英国的Gatsby计算神经科学中心）和以色列也建立了类似的中心。我们的第一项建议是在中国建立两个或多个理论神经科学中心，承担培养青年人才和协调计算神经科学发展的双重任务。中心可建在合适的大学、研究所，由政府和个人慈善家支持，可望成为全国此领域的枢纽、以及中国在神经科学与国际合作的平台。

第二项建议是支持计算神经科学的暑期学校。如上所述，在这样的暑期学校接受培训，对于不熟悉神经科学的理论学者转型以及希望学习建模和理论的实验工作者来说都至关重要。实际上，十年前中国就开设了一个国际暑期课程，迄今为止，该课程已经培训了大约270名研究生和博士后，并已得到国际上广泛认可（www.ccnss.org）。这样的暑期项目应该获得政府以及企业和私人赞助商的长期支持。

我们的第三项建议是启动新的基金项目。理论神经科学研究必须与实验紧密结合，而且理论神经科学项目的评审人员应包括来自非生命学科领域的专家。传统的项目评估体系难以满足这两个需要。基于这些考虑，美国国家科学基金会和美国国立卫生研究院联合发起了一个计算神经科学协作研究（CRCNS）计划。一个典型的CRCNS项目需要一个实验学家和一个理论学家共同申请。因此，理论学家不再仅仅基于发表的数据来建模，而是从“第一天”起与实验学家合作，从提出科学问题并设计实验、到完成该问题的研究。通过这种反复来回迭代的交流，理论和实验才能真正以一种互动的、双向的方式良性发展。在中国，这类项目的建立将对促进计算神经科学的发展产生关键的作用。目前，CRCNS已相继与德国、法国、以色列和日本建立了联合项目。一旦中国自己的基金项目建立起来后，未来可以很自然地考虑与CRCNS进行国际合作。

综上所述，我们在此表达在中国开展优化的计算神经科学学科建设的紧迫性，实现这个目标需要尽早规划。

1. BRAIN Initiative (2014): Working Group Report to the Advisory Committee to the Director, NIH. <https://braininitiative.nih.gov/sites/default/files/pdfs/brain2025_508c.pdf>
2. Levenstein, D *et al.* (2020) On the role of theory and models in Neuroscience. arXiv [https://arxiv.org/abs/2003.13825](https://arxiv.org/abs/2003.13825)
3. Wang X-J (2009) Introduction to Theoretical Neuroscience (in Chinese). Chapter 53, in Neuroscience, edited by Han Jiseng, Beijing University press, pp. 1004-1019. 汪小京 (2009) “理论神经科学导论”,《神经科学》 (第三版)，韩济生主编), 北京大学出版社, 第53章，1004-1019页.
4. Hodgkin, AL, Huxley, AF (1952). A quantitative description of membrane current and its application to conduction and excitation in nerve. *J. Physiol. (Lond.)*, 117, 500-544.
5. Hopfield, JJ (1982). Neural networks and physical systems with emergent collective computational abilities. *Proc. Natl. Acad. Sci. U.S.A.*, 79, 2554-2558.
6. Sejnowski, TJ, Koch, C, Churchland, PS (1988). Computational neuroscience. Science, 241, 1299-1306.
7. Abbott, LF (2008) Theoretical neuroscience rising. Neuron, 60, 3:489-95.
8. van Vreeswijk, C, Sompolinsky, H (1996). Chaos in neuronal networks with balanced excitatory and inhibitory activity. *Science*, 274, 1724-1726.
9. Wang X-J (2013) The prefrontal cortex as a quintessential “cognitive-type” neural circuit: working memory and decision-making. Principles of Frontal Lobe Function, Edited by DT Stuss and RT Knight, Second Edition, Cambridge University Press, pp. 226-248.
10. Wang X-J and Krystal J (2014) Computational Psychiatry. *Neuron* 84**,** 638-654.
11. Hassabis, D, Kumaran, D, Summerfield, C, Botvinick, M (2017). Neuroscience-Inspired Artificial Intelligence. *Neuron*, 95, 245-258.
12. BRAIN Initiative 2.0: From cells to circuits toward cures (2020) <https://braininitiative.nih.gov/strategic-planning/acd-working-groups/brain-initiative-20-cells-circuits-toward-cures>
13. 汪小京 (2010) “21世纪的中国计算神经科学展望”， 《科学时报》（Science Times）, 2010年8月25日. <http://news.sciencenet.cn/sbhtmlnews/2010/8/235983.html?id=235983>
14. 汪小京 (2015) “脑科学需要自己的牛顿“ ，科学网，2015年11月24日。<http://news.sciencenet.cn/htmlnews/2015/11/332429.shtm>
15. Poo, MM, Du, JL, Ip, NY, Xiong, ZQ, Xu, B, Tan, T (2016). China Brain Project: Basic neuroscience, brain diseases, and brain-inspired computing. *Neuron*, 92, 591-596.
